# MTBVAC: Attenuating the Human Pathogen of Tuberculosis (TB) Toward a Promising Vaccine against the TB Epidemic

**DOI:** 10.3389/fimmu.2017.01803

**Published:** 2017-12-15

**Authors:** Jesus Gonzalo-Asensio, Dessislava Marinova, Carlos Martin, Nacho Aguilo

**Affiliations:** ^1^Grupo de Genética de Micobacterias, Departamento Microbiología, Medicina Preventiva y Salud Pública, Universidad de Zaragoza, Zaragoza, Spain; ^2^CIBER Enfermedades Respiratorias, Instituto de Salud Carlos III, Madrid, Spain; ^3^Servicio de Microbiología, Hospital Universitario Miguel Servet, Instituto de Investigación Sanitaria Aragón (IIS Aragón), Zaragoza, Spain

**Keywords:** tuberculosis, Bacille Calmette-Guérin, live vaccines, ESAT6, CFP10, MTBVAC

## Abstract

Bacille Calmette-Guérin (BCG) is a live-attenuated strain of *Mycobacterium bovis* developed a century ago by repeated subculture. It remains the only vaccine against tuberculosis (TB) in use today, and it offers variable protection against the respiratory forms of TB responsible for transmission. The principal genetic basis for BCG attenuation is the loss of the region of difference 1 (RD1) that includes the genes codifying for production and export of the major virulence factor ESAT6. Today more than 13 TB vaccine candidates are in clinical evaluation. One of these candidates is MTBVAC, which is based on a rationally attenuated *Mycobacterium tuberculosis* clinical isolate belonging to modern lineage 4, one of the most widespread lineages among humans. MTBVAC conserves most of the T cell epitopes described for TB including the major immunodominant antigens ESAT6 and CFP10 of the RD1, deleted in BCG. After almost 20 years of discovery and preclinical development, MTBVAC is the only live attenuated vaccine based on a human pathogen that has successfully entered clinical trials as a preventive vaccine in newborns, aiming to replace BCG, and as a preventive vaccine in adolescents and adults (BCG-vaccinated at birth). Our recent preclinical studies have demonstrated that MTBVAC-induced immunity to ESAT6 and CFP10 correlate with improved efficacy relative to BCG encouraging exploration of these responses in human clinical trials as potential biomarkers and identification of these antigens as possible correlates of vaccine-induced protection. Such data would be extremely valuable as they would greatly accelerate clinical development to efficacy trials.

## Introduction

The current tuberculosis (TB) vaccine, Bacille Calmette-Guérin (BCG), is an attenuated strain of *Mycobacterium bovis* based on the etiologic agent of TB in cattle (1). BCG was introduced for the first time into clinical use almost a hundred years ago, when in 1921 it was given orally to an infant whose mother had died of TB a day after delivery. The infant showed no adverse events to vaccination with BCG and importantly, did not develop disseminated TB, the common form of disease at that time acquired mainly form unpasteurized milk of infected cows (2).

Today, BCG remains the only licensed vaccine against TB, given by the intradermal route of administration at birth. In infants, BCG is acknowledged to afford substantial protection against disseminated (miliary and meningeal) forms of TB (3, 4), whereas in adolescents and adults, lack of BCG efficacy against respiratory (pulmonary) TB, the most common form of the disease responsible for transmission, is considered to be a result of waning BCG immunity with time that is gradually lost 10 years after vaccination (5).

Tuberculosis has reached alarming proportions of 10.4 million incidence cases and 1.7 million deaths attributed to the disease as reported by the latest World Health Organization (6) global TB report 2017. Globally, some 50 million individuals are already latently infected with MDR *Mycobacterium tuberculosis* strains creating a remarkable resource for future cases of active TB with insufficient treatment options. Nevertheless, the WHO End TB Strategy has vowed to reduce TB morbidity by 90% and TB mortality by 95% by 2035 and recognizes the urgent need for more accessible diagnostic tools that are rapid and reliable, new less toxic and more efficacious antibiotics to shorten therapy and ultimately new vaccines to prevent pulmonary TB in order to achieve this ambitious goal (7).

There are a total of 12 candidates in the current clinical TB vaccine pipeline and they can be classified into three groups (2), including preventive pre- and postexposure subunit vaccines: viral or subunit vaccines that aim to boost immunity to BCG, killed or fractioned whole cell vaccines, and live-attenuated mycobacterial vaccines, which target either BCG-replacement at birth or prevention of TB in adolescents and adults.

Currently, the most advanced live-attenuated vaccine in clinical development is the recombinant BCG VPM1002, which is now entering efficacy trials aimed to prevent TB in infants with and without HIV exposure (8). The other candidate is the live-attenuated *M. tuberculosis* MTBVAC, which is the first vaccine of its kind to successfully enter human clinical evaluation in the history of human vaccinology. These two candidates could eventually replace BCG, if able to demonstrate improved safety and or greater efficacy than BCG when administered in newborns in high-burden TB-endemic settings.

**Figure 1:**
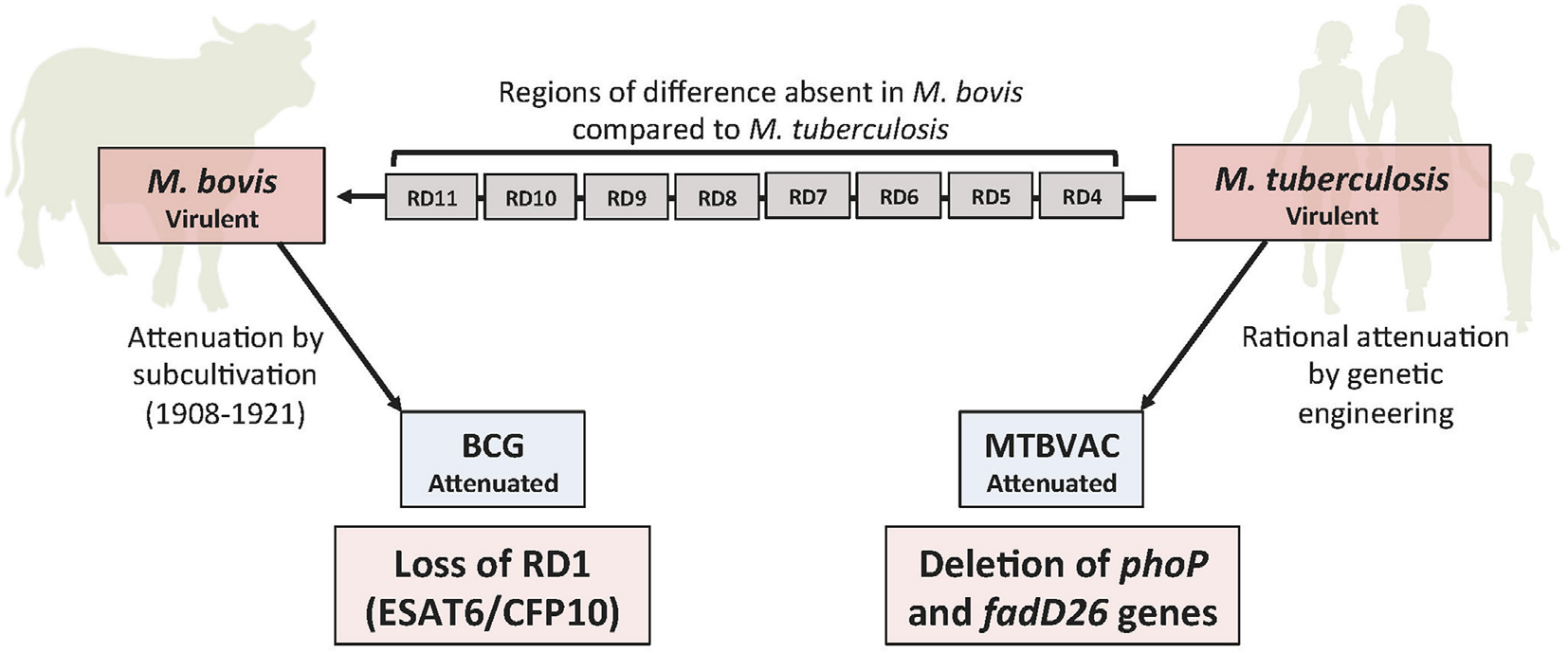
Genomic deletions between human and cattle tuberculosis pathogens. Eight regions of difference (RD) deleted in the bovine pathogen *Mycobacterium bovis* with respect to the human pathogen *Mycobacterium tuberculosis*. Repeated subcultivation of an *M. bovis* strain for 13 years (1908–1921), following classical Pasteur’s postulates, led to attenuation due to loss of RD1, giving rise to Bacille Calmette-Guérin (BCG). MTBVAC is the result of the rational attenuation of an *M. tuberculosis* clinical isolate by genetic deletions of the two independent virulence genes *phoP* and *fadD26*, following molecular Pasteur’s postulates for attenuated vaccines.

The main advantage of using rationally attenuated live *M. tuberculosis* as vaccine is that many genetic regions encoding important immunodominant antigens absent in BCG are still present in attenuated *M. tuberculosis*, while chromosomal deletions in virulence genes provide assurance for safety and genetic stability (Figure 1). These vaccines are expected to safely induce more specific and longer lasting immune responses in humans that can provide protection against all forms of the disease (9). This is the rationale that has been followed in the development of the live-attenuated MTBVAC.

## MTBVAC

MTBVAC was constructed by rational attenuation of the *M. tuberculosis* clinical isolate Mt103 (10), belonging to the modern *M. tuberculosis* Lineage 4, which together with Lineage 2 (Beijing strains) represent the most geographically widespread lineages of MTBC transmitted by the aerosol route between humans (11). Following the Geneva consensus safety requirements for progressing new live attenuated mycobacterial vaccines to clinical trials (12, 13), MTBVAC was constructed by generating two independent stable genetic deletions, without antibiotic resistance markers, in the genes *phoP* and *fadD26* encoding two major virulence factors (10). The gene *phoP* encodes the transcription factor of the two-component virulence system PhoPR and the gene *fadD26* participates in biosynthesis and export of phthiocerol dimycocerosates (PDIM), the main virulence-associated cell-wall lipids of *M. tuberculosis* (14–16). PhoP has been shown to regulate more than 2% of *M. tuberculosis* genome, most of which implicated in virulence (17, 18), and this regulation could be mediated through other transcription factors, e.g., WhiB6 of the ESX-1 system (19) or *via* non-coding RNAs, such as the recently described *mcr7* and its role in activating the TAT secretion system in MTBVAC leading to over secretion of major antigens such as those of the Ag85 complex (20). Other relevant virulence genes regulated by PhoP include lipid metabolism genes (e.g., *pks2, pks3*) involved in biosynthesis of the polyketide-derived acyltrehaloses (DAT, PAT) and sulfolipids, which are front-line lipid constituents of the cell wall thought to have a role in host immune modulation (21, 22). PhoP also regulates genes within ESX-1 (e.g., the *espACD* locus) implicated in the secretion of the major antigen and virulence factor ESAT6, so that *phoP*-mutants can produce but are unable to export ESAT6 (23).

Since 2001, rigorous preclinical safety, efficacy and immunogenicity studies of prototype SO2 and final vaccine construct MTBVAC were conducted in recognized animal models by independent (national and international) laboratories. Both MTBVAC and its prototype SO2 vaccine demonstrated to be at least as safe as BCG in SCID mice, and to confer greater or equivalent efficacy to BCG in different animal models (10, 24–27). SO2 conferred greater efficacy than BCG in a high-dose challenge, long-term survival experiment in guinea pigs (100% survival in SO2 group vs. 33% in the BCG group) (24). In guinea pigs, MTBVAC given as boost to BCG demonstrated significantly greater protection compared to BCG alone (27). In adult and newborn mouse models, MTBVAC conferred improved protection compared to BCG (25, 26) (Table 1).

**Table 1:** Comparison of Bacille Calmette-Guérin (BCG), MTBVAC, and H37Rv properties.

	BCG	MTBVAC	H37RV

Source	Virulent *M. bovis*	Virulent *M. tuberculosis*	Reference lab strain
Attenuation process	Subcultivation	Deletions in *phoP* and *fadD26* virulence genes	None
Number of epitopes	• •	• • •	• • •
Immunogenicity	•	• • •	• • •
Protection	• •	• • •	
Attenuation/safety	• • •	• • •	

The preclinical data with MTBVAC and SO2 provided a proof-of-concept to progress MTBVAC to first-in-human Phase 1 clinical evaluation. For this purpose, MTBVAC was developed and characterized as a freeze-dried vaccine product by the Spanish Biopharmaceutical company, Biofabri, in compliance with current Good Manufacturing Practices, fulfilling regulatory guidelines for assuring the quality, safety and stability, and efficacy of BCG freeze-dried vaccine (10).

The first-in-human Phase 1 trial designed to evaluate the safety, local tolerance and immunogenicity of three escalating dose of MTBVAC relative to BCG, was conducted in healthy, BCG-naive, HIV-negative adults. MTBVAC showed excellent safety and tolerability profile comparable to BCG. MTBVAC was at least as immunogenic as BCG, and at the same dose level as BCG, MTBVAC group showed greater frequency of polyfunctional CD4^+^ central memory T cells. This first Phase 1 trial marks a historic milestone in human vaccinology, as for the first time a live-attenuated *M. tuberculosis* vaccine has entered clinical trials showing excellent safety profile and differential immunogenicity profile compared to BCG supporting clinical development in high-burden TB endemic countries. Today MTBVAC follows two clinical development pathways, the primary supporting disease prevention at birth, as BCG-replacement strategy, and the secondary pathway supports prevention of pulmonary disease in adolescents and adults.

## ESAT6: A Double-Edged Sword

During the *in vitro* subcultivation process of BCG attenuation between 1908 and 1921, more than 100 genes were lost from BCG, relative to *M. tuberculosis* (28). Deletion of the region of difference 1 (RD1) region, which encodes the protein secretion system ESX-1, is considered the deletion responsible for BCG attenuation (28) (Figure 1). In a study by Copin et al. (29), where 1,530 experimentally verified human T cell epitopes were compared, it was demonstrated that 23% of the known MTBC T cell epitopes are absent in BCG. Despite their low molecular weight, the two major virulence factors ESAT6 and CFP10 encoded in the RD1 and absent in BCG, have been shown to contain the greatest number of human T cell epitopes recognized by the immune system (29). These data suggest the intensive interaction maintained throughout evolution between human hosts and these major RD1-encoded antigens of *M. tuberculosis*, which is likely a consequence of the crucial role of these proteins in virulence.

The role of ESAT6 in virulence has been demonstrated by experiments where complementation of BCG with RD1 restores full virulence (30), whereas deletion of RD1 in *M. tuberculosis* strains leads to a profoundly attenuated profile (31). ESAT6 has been described to interfere in different host–pathogen interaction processes: ESAT6 inhibits autophagy (32), a crucial cellular mechanism to eliminate intracellular pathogens, including mycobacteria (33). There exist strong evidences about the ability of ESAT6 to trigger cell death on host cells. A recent study demonstrated the contribution of the ESX-1 system to induce necrosis on infected neutrophils, in mechanism meditated by reactive-oxygen species (34); in addition, ESAT6 results also crucial for the bacteria to induce apoptosis on infected macrophages (35, 36), an event that provides the pathogen the capacity to spread from cell-to-cell maintaining an intracellular status (36), which might be important to establish early infection in the absence of initial recognition by the immune system (37, 38). During the last years, a role of ESAT6 in the escape of *M. tuberculosis* from the phagosome to the cytosol has been demonstrated to occur both *in vitro* and *in vivo* in mouse models of infection. This event has been considered relevant for infection outcome as strains unable to escape to cytosol have an attenuated phenotype (39–41). ESAT6 also stimulates the induction of type I interferon responses (42), which has been reported by some authors as detrimental for TB infection control (43).

Despite its great antigenic capacity, experiments in mice have shown that *esat6* and *cfp10*, as well as genes involved in their secretion, are highly expressed during different stages of lung infection (26, 44). In contrast, antigenic proteins such as those from the Ag85 complex, without an apparent role in virulence, have been shown to be downregulated upon infection (26, 45, 46). Antigen expression following infection might have an impact on the peptide repertoire presented by the MHC molecules from infected cells, which should be coated with peptides derived from the most represented proteins by the bacteria, including ESAT6 and CFP10, and not from those under expressed as Ag85. Indeed, human and mouse data indicate a higher degree of differentiation of ESAT6-specific T cells compare to Ag85B, due to the different antigen availability (47).

A classical approach for rational vaccine development has been the construction of vaccines that target the stimulation of a specific immune response against the most abundant proteins of the pathogen at each stage of infection. Thus, presence of a preexisting immunity against ESAT6 and CFP10 upon pathogen encounter with host might allowed rapid recognition of infected cells and would increase the probabilities to control infection before dissemination. In the particular case of BCG, which lacks of ESAT6 and CFP10, complementation of this strain with RD1 increases substantially its protective efficacy in different animal models (48, 49), suggesting that the deficient protection of BCG could rest on the absence of these major antigens. In addition, some subunit vaccines expressing ESAT6 have demonstrated good protection in mice and are currently under clinical evaluation (44). In contrast, the MVA85A vaccine demonstrated no improved protection compared to BCG in a phase IIb clinical trial, despite the good immunogenic profile shown (50). There are multiple reasons that could explain this failure, but it is plausible that the T cells reactive to Ag85A generated following boosting vaccination deficiently recognized infected cells due to the poor representation of Ag85A-derived peptides on cellular surface.

In the case of MTBVAC, this vaccine represents a unique candidate in the pipeline of vaccines currently under clinical evaluation, as it is the only one containing the whole antigen repertoire of *M. tuberculosis*, including the major antigens ESAT6 and CFP10 (28). Recently, we have demonstrated that an MTBVAC substrain mutant for these two proteins confers similar protection to BCG and lower than MTBVAC parental strain, suggesting a link between vaccine-induced protection and ESAT6/CFP10-mediated response, which could result in the definition of a biomarker for correlates-of-protection of MTBVAC (26).

Data available from MTBVAC-clinical trials indicated a significative CFP10-specific response in humans at six months post MTBVAC vaccination (26, 51). In this regard, it is crucial to elucidate the potential interference of MTBVAC in the quantiFERON (QFT) test used nowadays to discern infected individuals, as this assay is based on specific reactogenicity against ESAT6 and CFP10. Current MTBVAC clinical trials in TB endemic countries will be key to define this important question. In case of interference, we should be cautious with respect of the interpretation of the results. A direct translation of our preclinical data to humans could indicate that positive individuals to QFT induced by vaccination could be protected. The current practices where QFT-positive individuals are directly treated with isoniazid could be detrimental to identify these potential MTBVAC-mediated protection, as MTBVAC is a live vaccine, and therefore, sensitive to antibiotics. Interestingly, a recent study describes that mainly individuals with a high QFT value (higher than 4 IU/ml) have significantly a higher risk of develop active TB, whereas individuals with a value between 0.35 and 4 have no more probabilities than negative individuals (52). These data probably reveal the need to reconsider the cutoff of this assay in order to treat with isoniazid only individuals with a real risk to develop TB.

## MTBVAC Carries the Epitope Repertoire of *M. Tuberculosis*

Pioneering studies exploring the genomic basis behind BCG attenuation have identified several genetic regions present in *M. bovis* but deleted in BCG (1) (Figure 1). These deletions arose during subcultivation in the laboratory probably because the parental *M. bovis* strain was not subjected to the immune pressure of the host and hence it gradually lost virulence factors. BCG continued being passaged in the laboratory until lyophilization process was established, resulting in the emergence of different BCG “daughter” strains (53). These substrains differ in genome polymorphisms and show variable efficacy (54). Today, we know that BCG polymorphisms affect the synthesis of important virulence factors, such as the *phoPR* virulence regulator or PDIM (55–57) which might explain the different efficacy of BCG strains.

**Figure 2:**
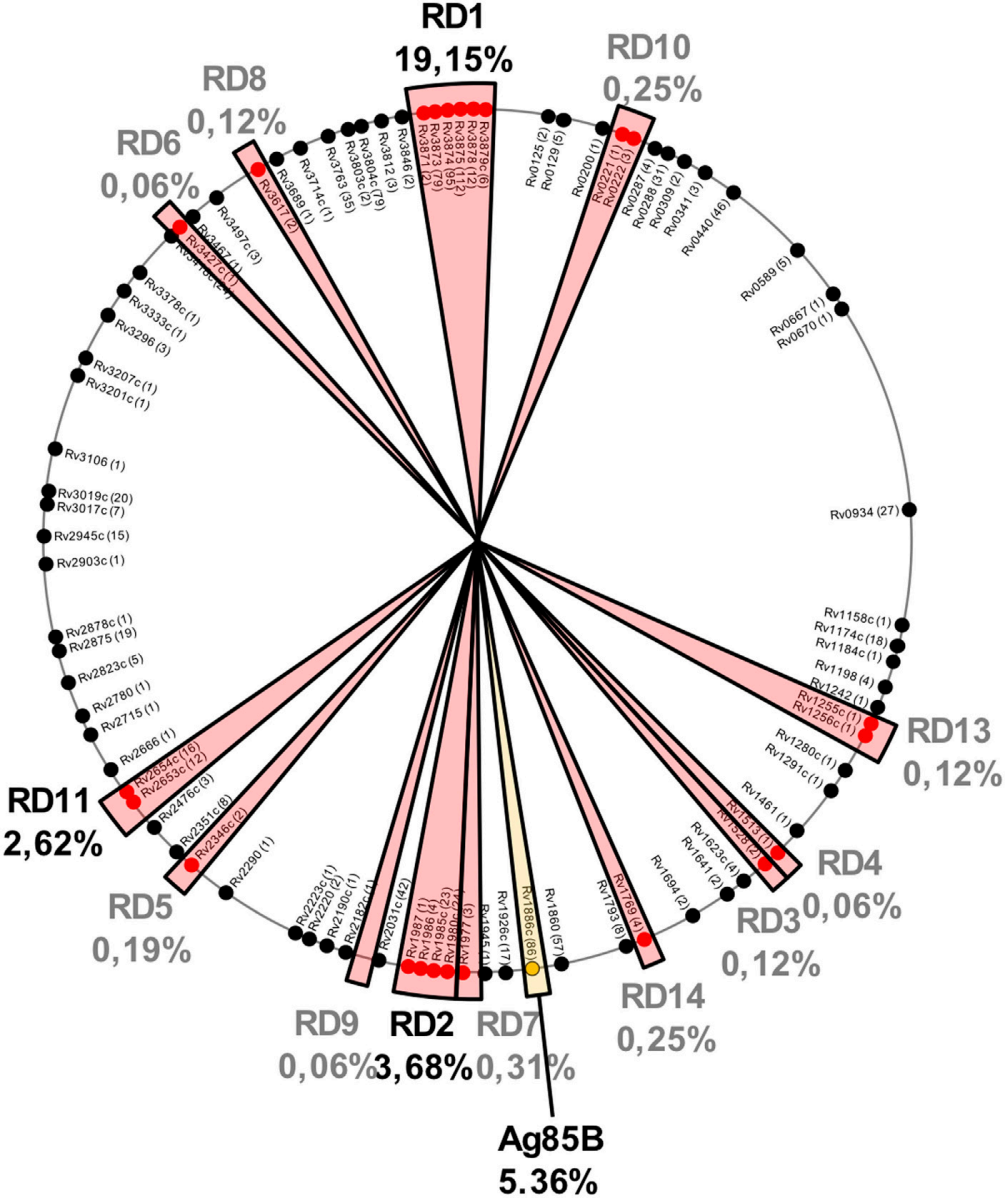
Localization of 1,603 experimentally demonstrated T cell epitopes (according to http://IEDB.org) in the *Mycobacterium tuberculosis* H37Rv chromosome. Those regions absent in Bacille Calmette-Guérin (BCG) but present in *M. tuberculosis* are shown by red sectors and the percentage of epitopes contained in each region is indicated. Three of these regions (RD1, RD2, and RD11) contain >25% of the total *M. tuberculosis* epitopes. It is also important to note that Ag85B (shown by a yellow sector) produced but not secreted by BCG also contains a high percentage of epitopes. Taken together, these results indicate that live attenuated vaccines based on the human pathogen, as MTBVAC, would present almost 40% more T cell epitopes than BCG.

It is important to remark that regions lost in BCG strains also contain potent antigenic proteins. After an updated analysis based on previous studies (29, 58), we found that of the 1,603 experimentally validated human T cell epitopes, 433 (27%) of them are located in RD regions and absent in BCG Pasteur (Figure 2). These epitopes are distributed as follows: RD1 307 epitopes (19.15%), RD2 59 epitopes (3.68%), RD11 42 epitopes (2.62%), RD 7 5 epitopes (0.31%), RD10 and RD14 4 epitopes each (0.5%), RD5 3 epitopes (0.19%), RD3, RD8, and RD13 2 epitopes each (0.36%), and RD4, RD6, and RD9 1 epitope each (0.18%). The vast number of epitopes absent in BCG is located in only five antigenic proteins: ESAT6 (112 epitopes), CFP10 (95 epitopes), and PPE68 (79 epitopes) within the RD1 region and MPT64 (24 epitopes) and Rv1985c (23 epitopes) within RD2 (Table 2).

**Table 2:** Summary table of described tuberculosis T cell-epitopes present in RD regions absent in Bacille Calmette-Guérin (BCG).

RD region	Rv number/gene name		# Epitopes	Sum
RD10	Rv0221		1	4
	Rv0222	*echA1*	3	
RD13	Rv1255c		1	2
	Rv1256c	*cyp130*	1	
RD4	Rv1513		1	1
RD3	Rv1582c		2	2
RD14	Rv1769		4	4
RD7	Rv1965	*yrbE3B*	1	5
	Rv1973		1	
	Rv1977		3	
RD2	Rv1979c		6	59
	Rv1980c	*mpt64*	24	
	Rv1984c		1	
	Rv1985c		23	
	Rv1986		4	
	Rv1987		1	
RD9	Rv2074		1	1
RD5	Rv2346c		2	3
	Rv2350c	*pIcB*	1	
RD11	Rv2645		13	42
	Rv2653c		12	
	Rv2654c		16	
	Rv2658c		1	
RD6	Rv3427c		1	1
RD8	Rv3617	*ephA*	2	2
RD1	Rv3871	*eccCb1*	2	307
	Rv3873	*ppe68*	79	
	Rv3874	*cfp10*	95	
	Rv3875	*esat6*	112	
	Rv3876	*espl*	1	
	Rv3878	*espJ*	12	
	Rv3879c	*espK*	6	
			Total	433

It has been described that Ag85B carries an F140L substitution predicted to affect protein stability (29). Moreover, our recent results demonstrated that Ag85B is absent in the secreted fraction of BCG but not of MTBVAC (26). Ag85B contains 86 epitopes which account for up to 5.36% of the total epitope content of *M. tuberculosis*. Remarkably, vaccination of C57BL/6 mice with BCG resulted in lack of Ag85B-specific response (26).

Of the whole epitope repertoire of the *M. tuberculosis* chromosome, BCG has lost 519 epitopes during its evolution distributed in 433 epitopes in RD regions and 86 epitopes in Ag85B. In other words, attenuated vaccines based on the human pathogen *M. tuberculosis* as MTBVAC, contains up to 48% more epitopes than BCG and consequently they are expected to produce a more robust stimulation of the immune system than BCG. It was previously demonstrated that MTBVAC-like vaccines exhibit a long-term maintenance of CD4^+^ T cell memory compared to BCG. Notably, this phenotype is specific of Ag85B since these experiments were performed in transgenic mice that exclusively respond to the p25 peptide of this protein (59).

MTBVAC not only produces all the antigens present in *M. tuberculosis* but it is also able to secrete some of these antigens more efficiently than other vaccines. We recently demonstrated that among the PhoP-regulated genes, the more tightly regulated region corresponds to a small RNA named *mcr7*. Consequently, *mcr7* is absent in *phoP* mutants such as the MTBVAC vaccine. Since *mcr7* exert a negative effect over translation of *tatC*, this protein involved in the TAT (Twin Arginine Translocation) secretion system exhibit higher levels in *M. tuberculosis phoP* mutants and results in more efficient secretion of TAT substrates (20). Among these, we found Ag85A (79 epitopes) and Ag85C (5 epitopes). Future experiments will demonstrate whether this enhanced antigenic secretion translate into more efficient immunity against these antigens.

Exploration of animal-adapted species of the MTBC such as *M. bovis* have revealed some epitope polymorphisms compared to *M. tuberculosis* (60) and this list is expected to increase as more animal isolates become sequenced. Two recent studies have demonstrated that these epitope polymorphisms result in differential immune responses (61) or MHC recognition (60). Consequently, even if the antigen repertoire from the human and the cow pathogen is highly similar, we can hypothesize that a vaccine for human use will benefit from stimulating the immune system with the pathogen from human origin.

## Concluding Remarks

In the current pipeline of TB vaccine candidates in clinical evaluation, MTBVAC is the only vaccine able to induce CFP10- and ESAT6-specific immune responses. What is more, our most recent results (26) suggest that these responses could be effective in protecting from pulmonary TB, which would have an evident impact on TB transmission. In support of this hypothesis, prospective cohort studies with individuals exposed to patients with active TB indicate that persons with latent TB infection (i.e., those reactive to CFP10 and ESAT6 stimulation) could be more protected against secondary *M. tuberculosis* infection than non-infected people (62). Thus, analysis of CFP10- and ESAT6-positive cells after vaccination in future clinical efficacy trials with MTBVAC would be highly valuable in the search of a possible biomarker of protection.

## Author Contributions

NA, DM, JG-A, and CM contributed equally in the elaboration of the manuscript.

## Conflict of Interest Statement

CM and JG-A are coinventors of the patent “tuberculosis vaccine” owned by the University of Zaragoza and licensed in exclusivity to Biofabri. CM and NA are coinventors of the patent “Inactivated tuberculosis vaccine” filled by the University of Zaragoza and Biofabri. The authors have no other relevant affiliations or financial involvement with any organization or entity with a financial interest in or financial conflict with the subject matter or materials discussed in the manuscript apart from those disclosed. The reviewer CH and handling editor declared their shared affiliation.
